# Complete Genome Sequence of Porcine Parvovirus 2 Recovered from Swine Sera

**DOI:** 10.1128/genomeA.01627-15

**Published:** 2016-01-28

**Authors:** F. S. Campos, M. Kluge, A. C. Franco, A. Giongo, F. P. Valdez, T. M. Saddi, W. M. E. D. Brito, P. M. Roehe

**Affiliations:** a Laboratório de Virologia, Instituto de Ciências Básicas da Saúde, Universidade Federal do Rio Grande do Sul, Porto Alegre, Rio Grande do Sul, Brazil; b Instituto do Petróleo e dos Recursos Naturais, Pontifícia Universidade Católica do Rio Grande do Sul, Porto Alegre, Rio Grande do Sul, Brazil; c Pontifícia Universidade Católica do Rio Grande do Sul, Faculdade de Biociências, Porto Alegre, Rio Grande do Sul, Brazil; d Instituto de Patologia Tropical e Saúde Pública, Universidade Federal de Goiás, Goiânia, Goiás, Brazil

## Abstract

A complete genomic sequence of porcine parvovirus 2 (PPV-2) was detected by viral metagenome analysis on swine sera. A phylogenetic analysis of this genome reveals that it is highly similar to previously reported North American PPV-2 genomes. The complete PPV-2 sequence is 5,426 nucleotides long.

## GENOME ANNOUNCEMENT

Porcine parvovirus (PPV) is a small nonenveloped virus associated with reproductive problems and is endemic in virtually all swine-producing regions worldwide (1). The viral genome is a single-stranded DNA molecule of approximately 5 kb (2). Porcine parvovirus 2 (PPV-2) was ﬁrst detected in 2001 and is probably distributed worldwide; however, links between PPV-2 and disease remain unclear (3).

Here, the complete sequence of a PPV-2 genome, identified in sera of Brazilian sows, is reported. Sera from ten clinically healthy, 5-months-old gilts (*Sus scrofa*) of the Large White breed were collected in Senador Canedo, Goiás, Brazil. Samples were pooled and viral particles were pelleted by centrifugation. The pellet was resuspended in Tris-EDTA and treated with DNase I (Roche) and RNase (Invitrogen). Viral genomes were extracted with a commercial kit (PureLink Viral RNA/DNA minikit) and amplified by random PCR (4). The products were purified and sequenced on an Ion Torrent platform with a 316 Ion chip. A total of 261,836 raw reads were generated and reduced to 126,661 after trimming with the Geneious version 8.0.2. From these, 18,862 reads were filtered by closest matching, where 95% of those fit within the family *Parvoviridae.* The reads were *de novo* assembled, with a mean coverage of at least 347×. A contig of 5,426 nucleotides (nt) comprised the full Brazilian PPV-2 (BrPPV-2) genome. Phylogenetic analyses performed with MEGA version 6.0 revealed that BrPPV-2 is closely related to genomes of North American PPV-2, with an overall 98.3 to 98.7% nt identity. The complete sequence of BrPPV-2 reveals two putative open reading frames (ORF): ORF1 (1,985 nt) and ORF2 (3,098 nt). In addition, two 14 nt-long palindromic sequences were identified, one at each extremity of the genome.

### Nucleotide sequence accession number.

The GenBank accession number is KM926355.
